# Radiotherapy modulates tumor cell fate decisions: a review

**DOI:** 10.1186/s13014-022-02171-7

**Published:** 2022-12-01

**Authors:** Haoran Chen, Zhongyu Han, Qian Luo, Yi Wang, Qiju Li, Lisui Zhou, Houdong Zuo

**Affiliations:** 1 Chengdu Xinhua Hospital, Chengdu, China; 2 School of Medical and Life Sciences, Chengdu University of Traditional Chinese Medicine, Chengdu, China

**Keywords:** Radiotherapy, Apoptosis, Necrosis, Necroptosis, Senescence, Mitotic catastrophe, Autophagy, Pyroptosis, Ferroptosis, Cuproptosis

## Abstract

Cancer has always been a worldwide problem, and the application of radiotherapy has greatly improved the survival rate of cancer patients. Radiotherapy can modulate multiple cell fate decisions to kill tumor cells and achieve its therapeutic effect. With the development of radiotherapy technology, how to increase the killing effect of tumor cells and reduce the side effects on normal cells has become a new problem. In this review, we summarize the mechanisms by which radiotherapy induces tumor cell apoptosis, necrosis, necroptosis, pyroptosis, ferroptosis, autophagy, senescence, mitotic catastrophe, and cuproptosis. An in-depth understanding of these radiotherapy-related cell fate decisions can greatly improve the efficiency of radiotherapy for cancer.

## Introduction

Cancer has always been a worldwide health problem and is the second leading cause of death after heart disease. In the United States, approximately 1.9 million new cancer cases are expected to appear in 2022 [1]. With the development of medical technology, radiotherapy (RT) has been widely applied to the treatment of cancer. X-rays were discovered in 1895 and radium and polonium were discovered by Marie Curie at the end of the century; thus, RT has been used for more than 100 years. It is estimated that approximately half of cancer patients use RT during treatment, which has led to a significant decrease in cancer mortality [2].

Cell fate decision (CFD) is an important mechanism of multicellular organisms and one of the foundations for maintaining biological stability. Compared with normal cells in the body, the biggest feature of tumor cells is the ability to escape death and proliferate indefinitely [3]. Therefore, inhibiting the proliferation ability of tumor cells, inducing tumor cell death, and eliminating tumor cells are the key steps to the treatment of tumors. Current studies have confirmed that RT could induce multiple tumor CFDs, such as programmed cell death (PCD): apoptosis, necroptosis, autophagy, pyroptosis, ferroptosis, and cuproptosis; non PCD: unregulated forms of necrosis, and non-lethal processes: senescence and mitotic catastrophe (Table 1). This article reviews how RT induces tumor CFDs, looking for breakthroughs in cancer mechanisms and treatments.

**Table 1:** Radiotherapy induces different cell fate decisions in tumor cells

Type of CFD	CFD characteristics	Features	Key factors	References
Apoptosis	PCD	Cell shrinkage, chromatin condensation, nuclear fragmentation, plasma membrane blebbing, production of apoptotic bodies	Caspase ↑; p53 ↑; Bcl-2 ↓; BAX ↑; PUMA ↑; NOXA ↑	[31, 32]
Necrosis	Non-PCD	DNA damage results in cell swelling, rupture of the plasma membrane, and outflow of cellular contents, followed by inflammation and damage	ATP ↓; NAD ↓; Ca^2+^ ↑	[13, 14, 36–38]
Necroptosis	PCD	A MLKL- dependent PCD with a necrotic morphology	Caspase-8 ↓; RIPK1 ↑; RIPK3 ↑; MLKL ↑	[47]
Pyroptosis	PCD	A GSDM-dependent PCD with a necrotic morphology	NLRP3 ↑; AIM2 ↑; caspase-1 ↑; GSDMD ↑; IL-18 ↑; IL-1β ↑	[51, 52, 55]
Ferroptosis	PCD	An iron-dependent lipid peroxidation-induced PCD with a necrotic morphology	Fe^2+^ ↑; SLC7A11-GSH-GPX4 ↓ PUFA-PL ↑; ACSL4 ↑; LPCAT3 ↑; NAD(P)H-FSP1-CoQ ↓	[64, 73, 74, 84]
Autophagy	PCD	Lysosome degrades autophagosomes that engulf cytoplasmic components, and can induce autophagic cell death	PI3K-AKT-mTORC1; p53; p73; E2F1	[94, 95]
Senescence	Non-lethal process	A state in which cells lose their ability to proliferate	p53-p21 ↓; p16^INK4a^-pRB ↓	[101, 105, 107]
Mitotic catastrophe	Non-lethal process	Abnormal nuclear morphology, multi nucleated, multi micro-nucleated giant cells	P53-p21 ↓; CDK1-cyclin ↑; CDK2-cyclin E/A ↑	[118–120]
Cuproptosis	PCD	An PCD dependent on copper and protein acylation in TCA cycle with a necrotic morphology	Cu^+^ ↑; FDX1 ↑; LIAS ↑; DLAT ↑	[124, 125]

ACSL4, acyl-CoA synthetase long-chain 4; BAX, Bcl-2-associated X protein; Bcl-2, B-cell chronic lymphocytic leukemia/lymphoma-2; CDK, cyclin-dependent kinase; CFD: cell fate decision; FDX1: ferredoxin 1; FSP1: ferroptosis suppressor protein 1; GSDM, gasdermin; LPCAT3, lysophosphatidylcholine acyltransferase 3; MLKL, mixed lineage kinase domain-like protein; NAD, nicotinamide adenine dinucleotide; PCD: programmed cell death; PUFA-PL, phospholipid containing polyunsaturated fatty acid chain; PUMA, p53 upregulated modulator of apoptosis; RIPK, receptor-interacting serine/threonine-protein kinase; SLC7A11, solute carrier family 7 member 11; TCA, tricarboxylic acid

## Radiotherapy and cell fate decisions

The purpose of RT is to stop tumor cell proliferation and induce tumor cell death [4]. RT does not kill cells immediately, and tumor cell death may persist for days or even months after RT [3]. When ionizing radiation acts on cells, it can directly act on the molecules of cells, damaging DNA (about 30–40%) [5, 6]; it can also ionize water in cells (about 80% of cells are water) to generate reactive oxygen species (ROS), indirect damage to DNA (about 60–70%) [5, 6]. Tumor cells are more susceptible to RT than normal cells due to their high replication rate and defects in DNA damage response (DDR) pathways (such as mutations in ATR or ATM) [7, 8]. DNA double-strand breaks (DSBs) are the most serious DNA damage. When DSBs are not repaired in time, they can lead to different cell fate decisions. Currently, known RT-induced CFDs include PCDs such as apoptosis, necroptosis, pyroptosis, ferroptosis, and cuproptosis; unregulated forms of necrosis; and non-lethal processes of senescence and mitotic catastrophe. Different tumor CFDs induced by RT are related to various factors such as tumor cell type, time and dose of radiation, and tumor microenvironment.

## Radiotherapy and apoptosis

Apoptosis is a type of PCD that was first defined in 1972 [9]. Apoptosis occurs during growth, development, and aging, maintains the homeostasis of tissue cells and can also be triggered under the influence of diseases or various pathological stimuli [10]. In the process of apoptosis, shrinkage of the cell, chromatin condensation, nuclear fragmentation, blebbing of the plasma membrane, and the production of apoptotic bodies can be seen [11]. Apoptotic cells are quickly engulfed by neighboring phagocytes, without causing inflammation [12]. Despite the lack of systematic studies of the dose–response of RT in different tumor cell lines, many studies have shown that apoptosis, as a low-immunogenic PCD, was more easily induced by low-dose RT (high-dose RT was more likely to induce necrosis) [7, 13, 14]. This suggests that low-dose RT is less immunogenic.

RT-induced apoptosis includes the intrinsic pathway and the extrinsic pathway [15]. In the intrinsic pathway, RT induces apoptosis mainly through a caspase-dependent pathway [16]. Caspases belong to the interleukin-1β-converting enzyme family of proteases [17]. At present, 14 subfamily members of the caspase family have been identified, and they are divided into 3 subfamilies according to the homology of the amino acid sequence: apoptosis activators: caspase-2, -8 and -9; apoptosis executioners: caspase-3, -6 and -7; and inflammatory mediators: caspase-1, -4, -5, -11, -12, -13 and -14 [17]. RT can alter mitochondrial membrane permeability through a caspase-dependent intrinsic pathway, increasing and releasing proapoptotic factors into the cytoplasm, thereby triggering a series of apoptotic cascades [18].

Ionizing radiation affects all organelles in the cell, and the most important effect is DNA damage, which can directly affect the proliferation ability of cells. After ionizing radiation damages cellular DNA, the tumor suppressor protein p53 can be rapidly activated by ataxia telangiectasia mutated (ATM), ataxia telangiectasia and Rad3-related (ATR) serine/threonine kinases [19]. p53 is a transcriptional regulator of apoptosis mediator proteins, such as B-cell chronic lymphocytic leukemia/lymphoma-2 (Bcl-2), Bcl-2-associated X protein (Bax), p53 upregulated modulator of apoptosis (PUMA), and NOXA [20]. Bcl-2 and Bax belong to the Bcl-2 protein family, which can control the permeability of the mitochondrial membrane, thereby regulating the release of cytochrome c [21]. In the Bcl-2 protein family, Bcl-2 inhibits apoptosis by controlling the activation of caspase proteases, while Bax promotes apoptosis [22]. PUMA and NOXA also belong to the Bcl-2 protein family and promote cell apoptosis. Overexpression of PUMA can cause a reduction in mitochondrial membrane potential and the release of cytochrome C; NOXA can activate caspase-9 to exert a proapoptotic effect [23]. Cytochrome C release into the cytoplasm interacts with apoptotic protease activating factor-1 (Apaf-1), ATP, and pro-caspase-9 to form a structure called "apoptosome", which activates the apoptosis activator caspase-9 and further activates the apoptosis executioners caspase-3, -6 and -7 [24].

The extrinsic pathway is mediated by death ligand-receptor-specific binding to form constructs, such as tumor necrosis factor-a (TNF-a)/TNF-related apoptosis-inducing ligand (TRAIL)-TNF receptor 1 (TNFR1) or Fas ligand (FasL)-Fas/CD95 receptor [15]. Death ligand-receptor binding forms a death-inducing signaling complex (DISC), which activates caspase-8 and further activates caspase-3, -6, and -7 [25]. The extrinsic pathway and the intrinsic pathway are closely related. CD95 and TRAIL2 contain p53 response elements that can be activated by RT-induced upregulation of p53, thereby activating caspase-8 [26].

Caspase-3 is a key enzyme in apoptosis and can activate various downstream substrates, such as the caspase-activated deoxyribonuclease (CAD)-ICAD complex, lamin A, fodrin, DNA-PK and poly(ADP-ribose) polymerase (PARP) [27]. CAD binds to its inhibitor ICAD in the nucleus in normal cells. Caspase-3 cleaves the CAD-ICAD complex, releases CAD, and degrades DNA [28]. Lamin A and fodrin are components of the cytoskeleton [29]. DNA-PK and PARP are involved in DNA repair [30]. When caspase-3 activates its substrates, cell changes occur, such as shrinkage, chromatin condensation, nuclear fragmentation, blebbing of the plasma membrane, and the production of apoptotic bodies, leading to apoptosis.

There are many studies on apoptosis in RT, such as the targeted therapies against apoptotic pathways. Studies have shown that hypofractionated RT induced miR-34a expression and promoted apoptosis in nasopharyngeal carcinoma cells through a p53-dependent pathway [31]; LncRNA CCAT2 inhibited p53 and increased radioresistance in colon cancer [32].

## Radiotherapy and unregulated forms of necrosis

RT can cause necrosis-like death of tumor cells [33]. Morphologically, necrotic cells swell, the plasma membrane ruptures, and the cellular contents flow out, subsequently leading to a cascade of inflammation and injury [34]. According to different mechanisms, CFDs with necrotic morphology can be classified into unregulated forms of necrosis (necrosis) and various types of PCDs, such as necroptosis, pyroptosis, ferroptosis, and cuproptosis [33]. Depletion of the adenosine triphosphate (ATP) pool and nicotinamide adenine dinucleotide (NAD) and an increase in intracellular Ca^2+^ can lead to cell necrosis after radiation-induced DNA damage [35]. RT-induced DNA damage in tumor cells can induce cell necrosis. High-dose RT is more likely to cause necrosis than low-dose RT [13, 14]. In addition, necrosis can also be secondary to apoptosis [14]. When cancer RT induces massive apoptosis, if the local phagocytic compartment is overwhelmed, the apoptotic cells are not phagocytosed in time, which can also cause secondary necrosis and cause an inflammatory response [36, 37]. Schildkopf et al. found that RT induced limited apoptosis in epithelial origin-tumor cells, but could stimulate necrosis when combined with hyperthermia [38].

## Radiotherapy and necroptosis

Necroptosis is a mixed lineage kinase domain-like protein (MLKL) -dependent PCD with a necrosis morphology, and it is also one of the RT-induced cell fate decisions [39, 40]. The activation of necroptosis is mostly related to the death receptor pathway[41]. Taking the TNF-α-TNFR signaling pathway as an example, when the TNF-α-TNFR1 complex forms, it recruits receptor-interacting serine/threonine-protein kinase 1 (RIPK1), TNF receptor-associated death domain (TRADD), cellular inhibitor of apoptosis protein 1 (cIAP1), cIAP2, and TNF receptor-associated factor 2 (TRAF2) to form prosurvival complex I [41]. Complex I can be deubiquitinated to form multiple complex II [42].

**Fig. 1:**
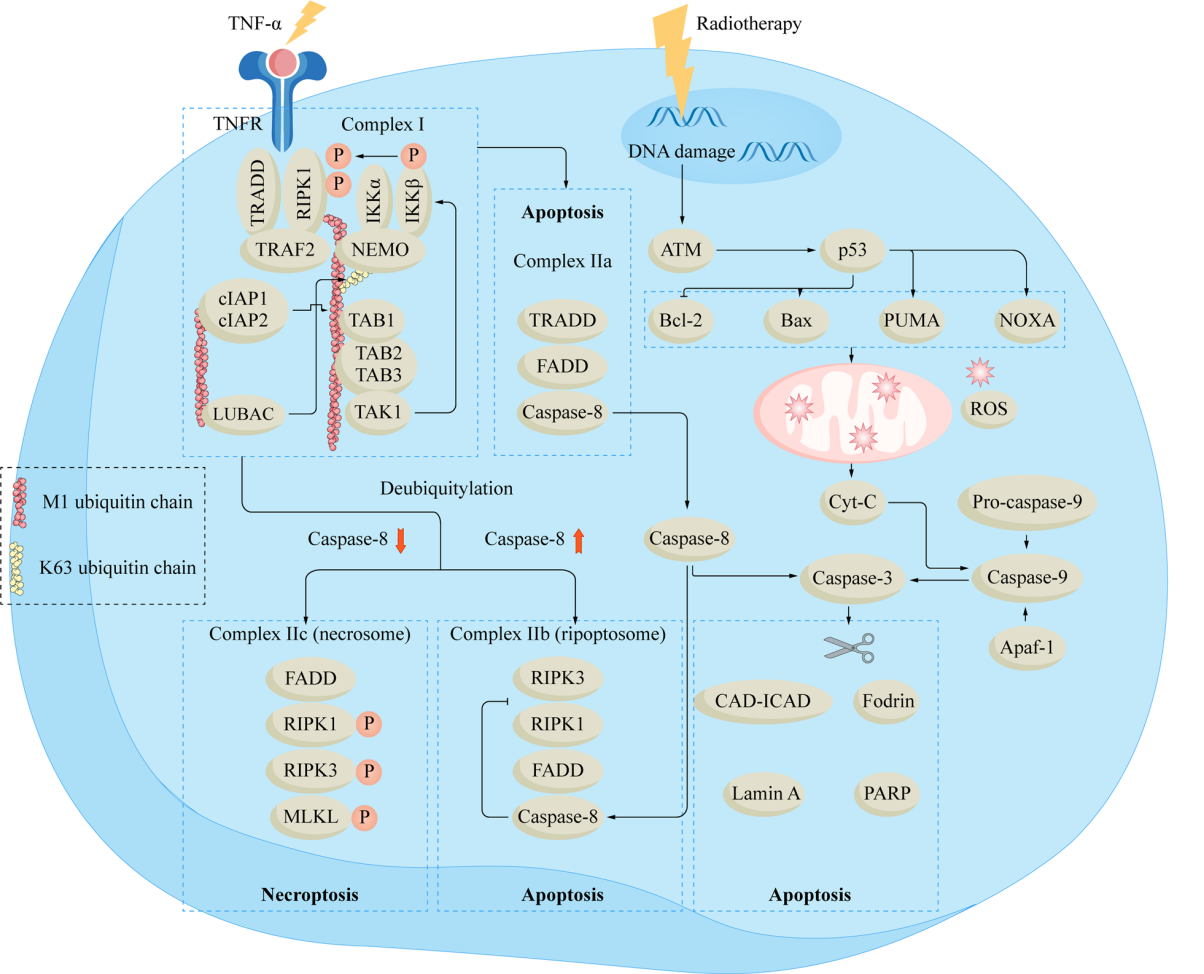
The mechanisms of RT-induced apoptosis and necroptosis in tumor cells. RT induces tumor cell apoptosis through the intrinsic pathway and the extrinsic pathway. In the intrinsic pathway, ionizing radiation damages DNA, leading to the activation of ATM and p53, which subsequently act on downstream molecules. These molecules alter the permeability of the mitochondrial membrane, leading to the release of cyt-c. Cyt-c interacts with Apaf-1 and pro-caspase-9 to activate caspase-9. Caspase-9 promotes the maturation of caspase-3 and leads to apoptosis. In the extrinsic pathway, TNF-αbinds to its receptor and subsequently recruits TRADD, cIAP1, cIAP2, TRAF2, and RIPK1 to form complex I. Complex I forms various complexes through deubiquitination. Complex IIa is composed of TRADD, FADD, and caspase-8, which can lead to the activation of caspase-8 and activate caspase-3 to promote apoptosis. Necroptosis is associated with the activity of caspase-8. When caspase-8 is activated, it can form complex IIb with RIPK1, RIPK3, and FADD, inactivate RIPK1 and RIPK3, and eventually lead to apoptosis; when caspase-8 is inactivated, complex IIc is formed. RIPK1 activates RIPK3, which recruits and activates MLKL. MLKL oligomerizes and changes the permeability of cell membranes, promoting necroptosis. Apaf-1: apoptotic protease activating factor-1; CAD: caspase-activated deoxyribonuclease; cIAP: cellular inhibitor of apoptosis protein; cyt-c: cytochrome-c; FADD: Fas-associating protein with a novel death domain; IKK: inhibitor of NF-κB (IκB) kinase; MLKL: mixed lineage kinase domain-like protein; RIPK: receptor-interacting serine/ threonine-protein kinase; RT: radiotherapy; TRADD: TNF receptor-associated death domain; TRAF2: TNF receptor-associated factor 2

Different complex II is associated with apoptosis or necroptosis. Complex IIa consists of TRADD, Fas-associating protein with a novel death domain (FADD), and caspase-8, which can activate caspase-8 and promote apoptosis [43]. RIPK1 and RIPK3 play important roles in necroptosis, and caspase-8 is closely related to the occurrence of necroptosis. When caspase-8 is activated, it forms complex IIb with RIPK1, RIPK3, and FADD, which cleaves RIPK1 and RIPK3, causing their inactivation and ultimately leading to apoptosis [44]. Conversely, the activity of caspase-8 may be inhibited with increasing radiation dose, as in glioblastoma [39, 45]. At this point, FADD, RIPK1, and RIPK3 form complex IIc, also known as necrosome, which subsequently induces necroptosis [39, 43, 45]. RIPK1 can recruit and activate RIPK3 to form necrosomes. RIPK3 can recruit MLKL and promote its phosphorylation [46]. MLKL then oligomerizes and migrates to the cell membrane, changing the permeability of the cell membrane and promoting necroptosis. Yang et al. found that ablation of caspase-8 could promote RT-induced necroptosis, increase the expression of MLKL, and improve the sensitivity of tumor cells MC38, A54, and B16-SIY to RT [47]. Figure 1 shows the mechanisms of RT-induced apoptosis and necroptosis in tumor cells.

## Radiotherapy and pyroptosis

Pyroptosis is an inflammatory PCD mediated by the GSDM family [48]. GSDM family members GSDM A-E have two characteristic conserved domains: N-terminal pore-forming domain (N-PFD) and C-terminal repression domain (C-RD) [49]. Under physiological conditions, C-RD interacts with N-PFD and inhibits N-PFD activity [49]. When cells are stimulated, the structure can be cleaved by caspase-dependent or -independent pathways and release cytotoxic N-PFD [50]. N-PFD oligomerizes in the membrane and forms membrane pores, causing the release of the inflammatory mediators IL-1β and IL-18 and pyroptosis [50].

RT can induce tumor cell pyroptosis through the inflammasome pathway and the non-inflammasome pathway. In the inflammasome pathway, RT can activate inflammasome AIM2 and NLRP3, recruit apoptosis-associated speck-like protein containing CARD (ASC) and pro-caspase-1, and activate caspase-1 [51–53]. Caspase-1 subsequently cleaves GSDMD and forms the GSDMD-N pore. Caspase-1 also cleaved pro-IL-18 and pro-IL-1β, promoting IL-18 and IL-1β maturation and release through the GSDMD-N pore [54]. Han et al. found that in a mouse model of colorectal cancer, RT could simultaneously activate the inflammasomes AIM2 and NLRP3 to trigger pyroptosis [51]. Their study showed that caspase-1^−/−^ mice and AIM2^−/−^NLRP3^−/−^ mice were radioresistant, while AIM2^−/−^ mice and NLRP3^−/−^ mice were similar to wild-type mice [51]. Zhang et al. showed that RT in lung adenocarcinoma could induce tumor cell pyroptosis and exert a therapeutic effect [52]. In their study, RT damaged tumor cell DNA, activated AIM2, and subsequently induced caspase-1-GSDMD pathway-mediated pyroptosis, and reduced tumor volume and weight. Radiation dose-dependently downregulated circNEIL3 and activated AIM2 via the circNEIL3/ miR-1184/ PIF1 axis, triggering pyroptosis. In addition, overexpression of circNEIL3 inhibited caspase-1 and reduced the therapeutic effect of RT [52].

**Fig. 2:**
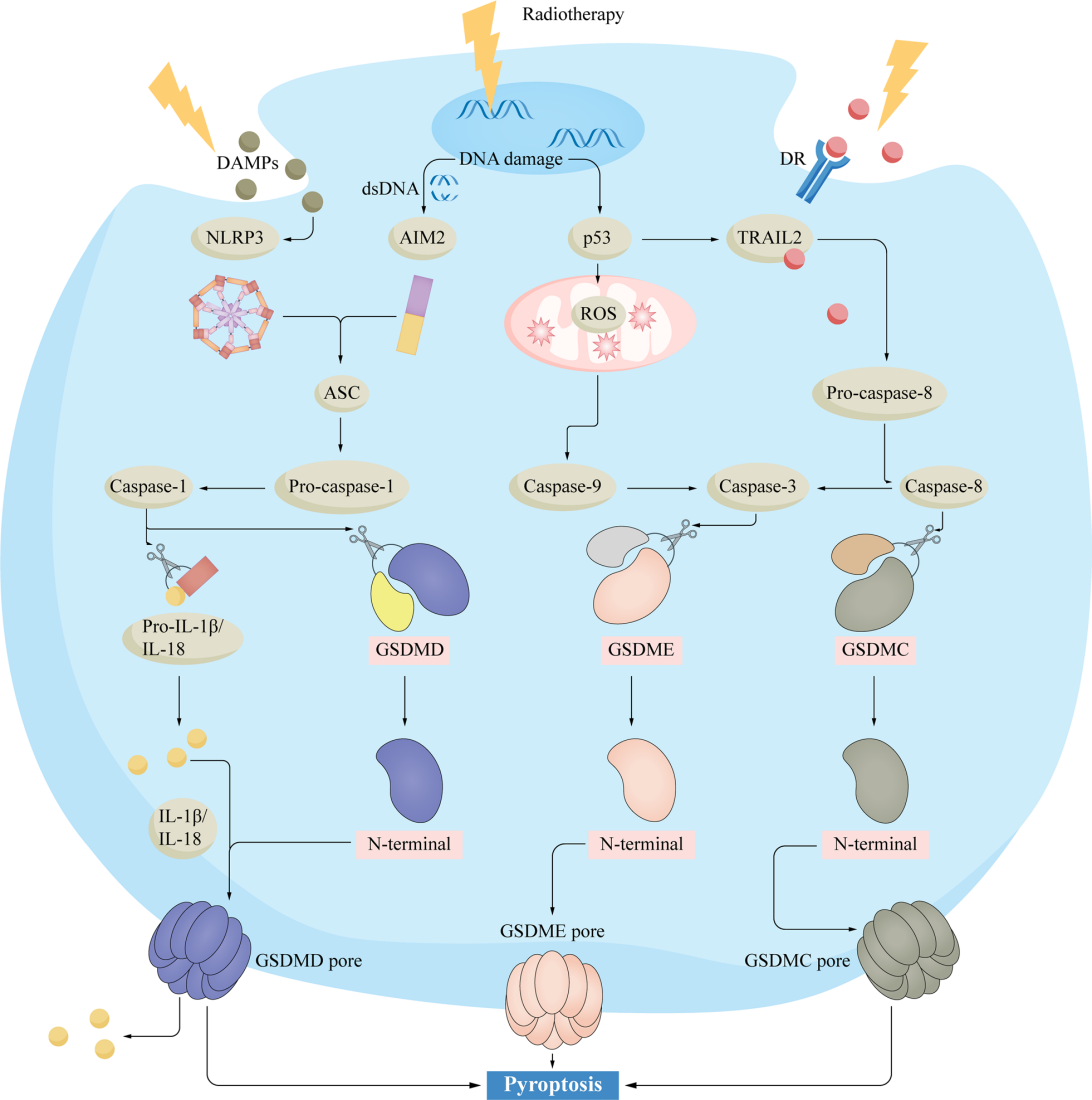
The mechanisms of RT-induced pyroptosis in tumor cells.RT-induced pyroptosis in tumor cells through the inflammasome pathway and the non-inflammasome pathway. In the inflammasome pathway, NLRP3 and AIM2 recruit ASC and pro-caspase 1 in response to RT, leading to the activation of caspase-1. Caspase-1 cleaves GSDMD, exposes N-PFD, and forms pores on the plasma membrane; caspase-1 cleaves pro-IL-1β and pro-IL-18, leading to maturation of the inflammatory cytokines IL-1β and IL-18 and released from GSDMD wells. In the non-inflammasome pathway, RT activates caspase-3 and -8 through the intrinsic pathway and the extrinsic pathway. Caspase-3 cleaves GSDME and caspase-8 cleaves GSDMC, forming GSDME and GSDMC pores in the plasma membrane, respectively, and inducing tumor cell pyroptosis. ASC: adaptor apoptosis-associated speck-like protein containing a CARD; DAMP: damage-associated molecular pattern; DR: death receptor, GSDM: gasdermin; N-PFD: N-terminal pore-forming domain; RT: radiotherapy; TRAIL2: TNF-related apoptosis-inducing ligand 2

Radiation also induces pyroptosis through the non-inflammasome pathway. GSDMs can be cleaved by a variety of apoptosis-related caspases and play a role in the transition from apoptosis to pyroptosis [48]. Tan et al. found that the resistance of colorectal cancer to RT was related to the low expression of GSDME, and RT could induce pyroptosis through the caspase-3-GSDME pathway after upregulation of GSDME [55]. In addition, despite the lack of relevant studies, the caspase-8-GSDMC pathway may also play a role in RT, as it has been shown that PD-L1 could mediate the conversion of cancer cell apoptosis to pyroptosis by GSDMC [56]. Figure 2 shows the mechanisms of RT-induced pyroptosis in tumor cells.

## Radiotherapy and ferroptosis

Ferroptosis was proposed in 2012 and is a new PCD different from apoptosis, necroptosis, and pyroptosis [57]. Increased mitochondrial membrane density, reduced mitochondrial cristae, and mitochondrial atrophy can be seen during ferroptosis [58]. Its pathogenesis is closely related to the accumulation of ROS and iron-dependent lipid peroxidation [59]. In cells, unstable iron free radicals can generate oxygen free radicals through the Fenton reaction to promote lipid peroxidation; iron also promotes ferroptosis as a cofactor in enzymes that promote lipid oxidation [60]. Ferroptosis plays an important role in RT-induced CFD.

The SLC7A11-GSH-GPX4 pathway plays an important role in the defense against ferroptosis. Glutathione peroxidase 4 (GPX4) can play an important role in inhibiting lipid peroxidation. GPX4 converts reduced glutathione (GSH) to oxidized glutathione (GSSG) and lipid hydroperoxide (L-OOH, toxic) to lipid alcohol (L-OH, no toxicity), thereby promoting the decomposition of H_2_O_2_ and inhibiting ferroptosis [61]. The rate-limiting precursor of the antioxidant GSH is cysteine, which is formed by the reduction of cysteine [62]. Intracellular cystine import is controlled by the cystine/glutamate antiporter (system X_c_-), and a core component of this system is solute carrier family 7 member 11 (SLC7A11) [63]. Inhibition of the SLC7A11-GSH-GPX4 pathway can lead to the accumulation of lipid peroxides and promote ferroptosis. Radiation damage to cellular DNA leads to an increase in ATM. Studies have shown that ATM could inhibit SLC7A11, reduce cystine import, and promote ferroptosis [64]. Meanwhile, p53 activation after DNA damage may play a dual role in the SLC7A11-GSH-GPX4 pathway. On the one hand, p53 can inhibit the transcription of SLC7A11 and promote ferroptosis [65]; on the other hand, activated p53 can upregulate p21, maintain GSH levels during stress, and inhibit ferroptosis [66]. p21 can also inhibit ferroptosis through a p53-independent pathway, possibly related to CDKs [67]. ROS generated by RT can also bind to GSH and then convert it to GSSG, thereby promoting ferroptosis [68].

Polyunsaturated fatty acids (PUFAs) are important for the normal physiological function of cells and can maintain the fluidity of cell membranes [69]. Due to the presence of bis-allylic in PUFAs, PUFAs are prone to lipid peroxidation, which is also required in ferroptosis [58].

During ferroptosis, acyl-CoA synthase long-chain 4 (ACSL4) and lysophosphatidylcholine acyltransferase 3 (LPCAT3) are key regulators and rate-limiting factors. ACSL4 can catalyze PUFA to form the corresponding acyl-coenzyme A (CoA) derivative PUFA-CoA [70]. PUFA-CoA is processed to form lysophospholipids (LysoPLs) and then is combined with phospholipids (PLs) by LPCAT3 to form PUFA-PLs [71]. PUFA-PLs are highly prone to peroxidation due to the presence of bis-allylic moieties. When the ACSL4 and LPCAT3 genes are knocked out, the synthesis of PUFA-PLs is reduced, and ferroptosis is suppressed [72]. In radiotherapy, the absorption of ionizing radiation by water results in ROS production, and ROS subsequently act on PUFAs, leading to lipid peroxidation in a dose-dependent manner [73]; meanwhile, although the mechanism remains unclear, radiotherapy can induce ACSL4 expression to promote PUFA-PL synthesis and ferroptosis [73].

**Fig. 3:**
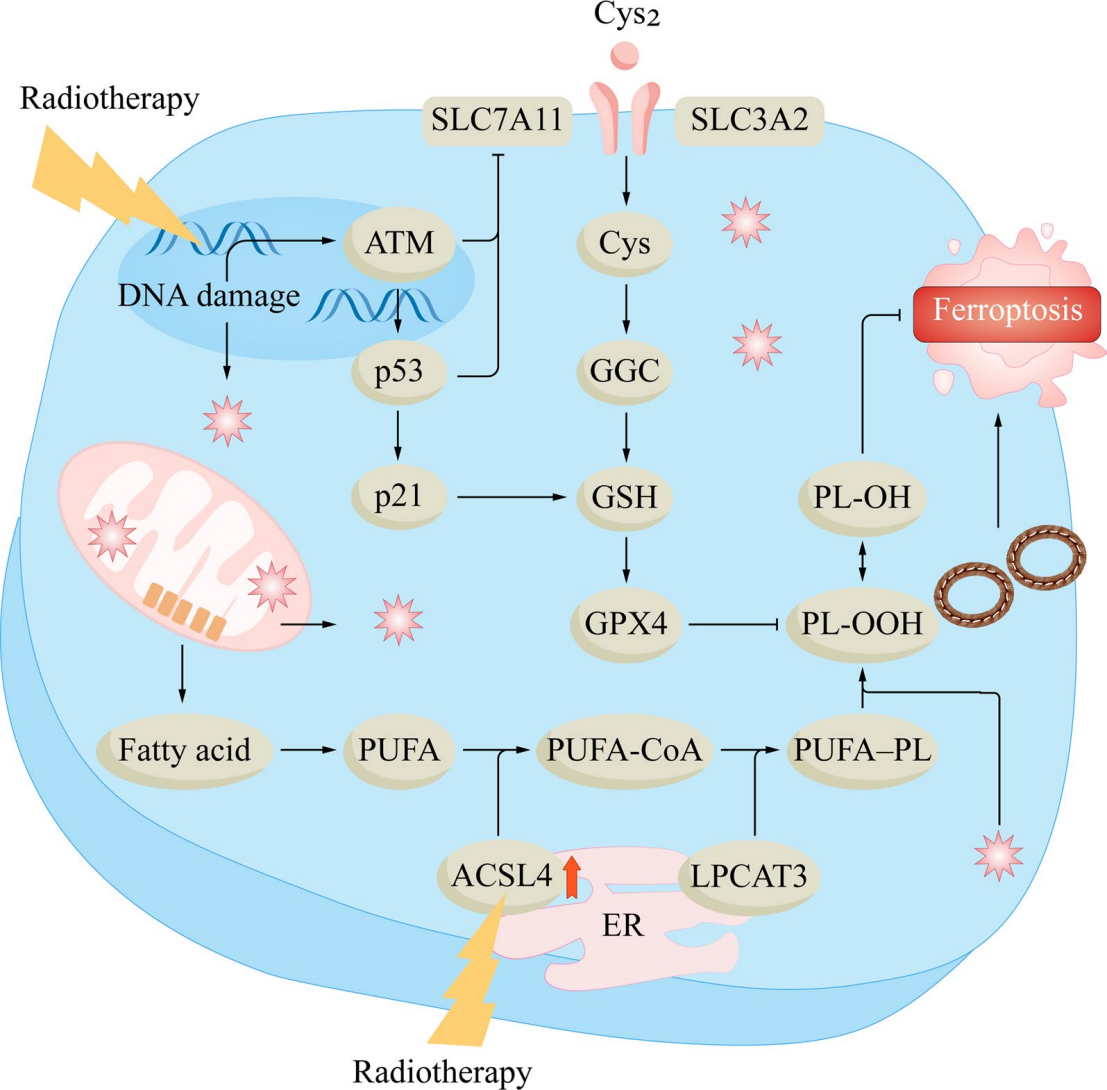
The mechanisms of RT-induced ferroptosis in tumor cells. Intracellular cystine import is controlled by the cystine/glutamate antiporter (System X_c_-), with SLC7A11 being the core component. Inhibition of the SLC7A11-GSH-GPX4 pathway leads to accumulation of lipid peroxides and promotes ferroptosis. Damage to cellular DNA by radiation leads to increased ATM. ATM has been shown to inhibit SLC7A11, reduce cystine import, and promote ferroptosis. Also, p53 activation following DNA damage may play a dual role in the SLC7A11-GSH-GPX4 pathway. On the one hand, p53 can inhibit SLC7A11 transcription and promote ferroptosis; on the other hand, activated p53 can up-regulate p21, maintain GSH levels during stress, and inhibit ferroptosis. During ferroptosis, ACSL4 and LPCAT3 are critical regulators and rate-limiting factors. ACSL4 can catalyze PUFA to generate PUFA-CoA. PUFA-CoA is processed to form lysophospholipids, which are then combined with phospholipids (PLs) by LPCAT3 to form PUFA-PLs. In RT, ROS can act on PUFAs and lead to lipid peroxidation in a dose-dependent manner. In addition, RT can induce ACSL4 expression, promote PUFA-PLs synthesis, and ultimately lead to ferroptosis. ACSL4, acyl-CoA synthetase long-chain 4; Cys2, cystine; ER, endoplasmic reticulum; GGC, γ-Glutamylcysteine; LPCAT3: lysophosphatidylcholine acyltransferase 3; PLOOH, phospholipid hydroperoxide. PUFA, polyunsaturated fatty acid; PUFA-PL, phospholipid containing polyunsaturated fatty acid chain; RT: radiotherapy; SLC7A11: solute carrier family 7 member 11

RT-induced ferroptosis may also be associated with other signaling pathways [75, 76]. In addition to the effect of p53 on the SLC7A11-GSH-GPX4 pathway, it may induce or inhibit ferroptosis in other ways. p53 can induce the activation of mouse double minute 2 (MDM2) [77]. Studies have shown that MDM2 could regulate lipid metabolism and ferroptosis suppressor protein 1 (FSP1) to promote ferroptosis [78]. FSP1 is a key factor in the NAD(P)H-FSP1-ubiquinone (CoQ) pathway. FSP1 can consume NAD(P)H, reducing CoQ to CoQH2. CoQH2 can bind to lipophilic free radicals and inhibit ferroptosis [79]. The NAD(P)H-FSP1-CoQ pathway is a GPX4-independent ferroptosis defense pathway [80]. Increased ROS during RT can induce the nuclear factor erythroid-2 related factor 2 (Nrf2)- heme oxygenase-1 (HO-1) pathway. The role of the Nrf2-HO-1 pathway in ferroptosis is also two-sided. Feng et al. found that Nrf2 could activate SLC7A11 to inhibit ferroptosis, and reduce the radiosensitivity of esophageal squamous cell carcinoma [74, 81]; Ruiran Wei et al. found that the activated Nrf2-HO-1 pathway could increase the level of Fe2^+^ and induce ferroptosis in colorectal cancer cells [82]. Figure 3 shows the mechanisms of RT-induced ferroptosis in tumor cells.

Induction of ferroptosis in tumor cells is one of the ways of radiosensitization. For example, [83] pancreatic cancer and renal cell carcinoma are sensitive to RT, which may be related to their dependence on cystine uptake [73, 84]; inhibition of SLC7A11 and promotion of ferroptosis can increase the radiosensitivity of esophageal squamous cell carcinoma [74]. Ferroptosis is also a new synergistic point for RT and immunotherapy. Lang et al. found that RT-activated ATM and immunotherapy-activated CD8^+^ T cells synergistically aligned SLC7A11 to enhance tumor cell ferroptosis and improve treatment outcomes [64].

## Radiotherapy and autophagy

Autophagy is a pathway by which cells engulf cytoplasmic components through autophagosomes and present them to lysosomes for degradation to maintain homeostasis and health [85]. During autophagy, characteristic double-membrane vesicles can be seen. The phosphatidylinositol 3-kinase (PI3K)- Protein Kinase B (AKT)- mechanistic target of rapamycin complex 1 (mTORC1) pathway is important for regulating autophagy [86]. Many studies have shown that a variety of drugs can inhibit the expression of members of the PI3K-AKT-mTORC1 pathway and induce autophagy [87–89].

RT can induce two types of autophagy in tumor cells: protective autophagy and autophagic cell death [90]. Genes, such as p53, p73, and E2F1, play an important role in this process [91–93]. RT can cause DNA damage through direct and indirect means while generating large amounts of ROS, which can induce cytoprotective autophagy. Activated protective autophagy can remove damaged mitochondria, reduce cellular oxidative stress, and improve tumor cell resistance to radiation [90]. Mo et al. found that reduced Rad51 expression could inhibit autophagy and increase radiosensitivity in nasopharyngeal carcinoma cells [94]. Wang et al. found that SMAD4 gene mutation could increase the radioresistance of pancreatic cancer by promoting autophagy [95].

On the other hand, some studies have found that the self-renewal ability of tumor cells is destroyed after ionizing radiation, and the mitosis of cells is blocked, which stimulates excessive autophagy of cells, resulting in autophagic cell death [90]. Djuzenova et al. [96] found that the radiosensitivity of glioblastoma increased after inhibition of the PI3K-AKT-mTORC1 pathway and induction of autophagy. Palumbo et al. found that the glioblastoma cell line T98G exhibited high radiosensitivity after treatment with an ionizing radiation-temozolomide combination, which was associated with the activation of autophagy [97]. After autophagy inhibition, the radiosensitivity of T98G cells decreased, indicating that RT played an anticancer effect through autophagic cell death. After induction of autophagy with rapamycin, the radiosensitivity of T98G was also enhanced [97].

Different forms of autophagy occur during RT, and the strength of autophagy activation and the targets of autophagy are key to determining whether autophagy promotes survival or death during RT-induced CFDs. An in-depth understanding of different forms of autophagy will be key to the development of individualized tumor therapy in the future.

## Radiotherapy and senescence

Senescence is a common non-lethal CFD induced by tumor RT [98]. Senescence refers to a state in which cells lose their ability to proliferate [99]. Senescent cells remain metabolically active, but cell cycle arrest [98]. Senescent cells show flattening, vacuolization, and altered DNA structure. Radiation-induced senescence is mainly controlled by the activation of the p53-p21 pathway and the p16^INK4a^-retinoblastoma protein (pRB) pathway [14, 100]. After ionizing radiation damages tumor cell DNA, activated p53 upregulates the expression of p21, a cyclin-dependent kinase (CDK) inhibitor that inhibits the expression of CDK-cyclin complexes [101–103]. Inhibition of CDK1-cyclin A complex arrests cells in the G2/M phase [101, 103]. Inhibition of CDK2-cyclin E/A complex and CDK4-cyclin D complex promotes dephosphorylation of pRB and arrests cells in the G1/S phase [101, 103]. Ionizing radiation-induced elevation of intracellular ROS and protein kinase C (PKC) can also increase p16^INK4a^ expression, inhibit CDK-cyclin complexes and promote pRB dephosphorylation, leading to tumor cell senescence [104].

Senescence plays an important role in tumor RT. Senescence can stop the proliferation of tumor cells, especially in tumor cells that have received non-lethal doses of radiation [101]. At an early stage, senescent cells can promote the recruitment of immune cells through senescence-associated secretory phenotype (SASP) [99]. Meanwhile, in some tumors (such as lung cancer and glioblastoma), RT-induced tumor cell senescence will preferentially induce apoptosis [101, 105]. However, in the later stages of RT, the accumulation of senescent cells may adversely affect prognosis, as the immune microenvironment generated by SASP may promote tumor recurrence [101, 106] The high recurrence rate of some cancers after RT may be related to the accumulation of senescent cells [101, 107]. For example, patients with non-small cell lung cancer who received stereotactic body RT had higher tumor recurrence rates than patients who received surgery at the same stage [101, 107, 108].

## Radiotherapy and mitotic catastrophe

Mitotic catastrophe usually occurs during or after abnormal mitosis. Due to abnormal cell mitosis, cells undergo atypical chromosome segregation and cell division, resulting in giant cells with abnormal nuclear morphology, multiple nuclei, and multiple micronuclei [109]. The mechanism of mitotic catastrophe is related to the promotion of mitosis and centrosome hyperamplification [110]. The role of mitotic catastrophe in anticancer therapy increases as tumor cells become more resistant to the induction of intrinsic apoptosis [98]. Like senescence, mitotic catastrophe is considered a non-lethal process, and the mitotic catastrophe does not always lead to apoptosis or necrosis, but sometimes to cellular senescence [98].

p53 is an important tumor suppressor gene that is mutated or inactivated in many tumors. p53 plays an important role in the regulation of mitotic checkpoints. Tumor cells readily undergo mitotic catastrophe after RT-induced DNA damage. Fragkos et al. found that following p53 inactivation, RT neither activated p21 nor inhibited CDK1-cyclin A complex, resulting in G2/M checkpoint inactivation and premature entry into mitosis [111]. p53 is also important for DNA repair, and cells that enter mitosis prematurely will carry unrepaired RT-induced DNA damage, promoting mitotic catastrophe.

Mitotic catastrophe is also associated with centrosome hyperamplification. When p53 is inactivated, RT does not upregulate p21 and does not inhibit the activity of the CDK2-cyclin E/A complex. The activity of the CDK2-cyclin E/A complex is critical for the initiation of centrosome amplification [112]. Studies have shown that centrosome hyperamplification can be seen when p53 is inactivated [113]. Centrosomes can form bipolar mitotic spindles during mitosis, and hyperamplified centrosomes can lead to multipolar spindles during mitosis. Multipolar spindles lead to abnormal chromosome segregation and produce giant cells with abnormal nuclear morphology, multiple nuclei, and multiple micronuclei, leading to mitotic catastrophe [109].

**Fig. 4:**
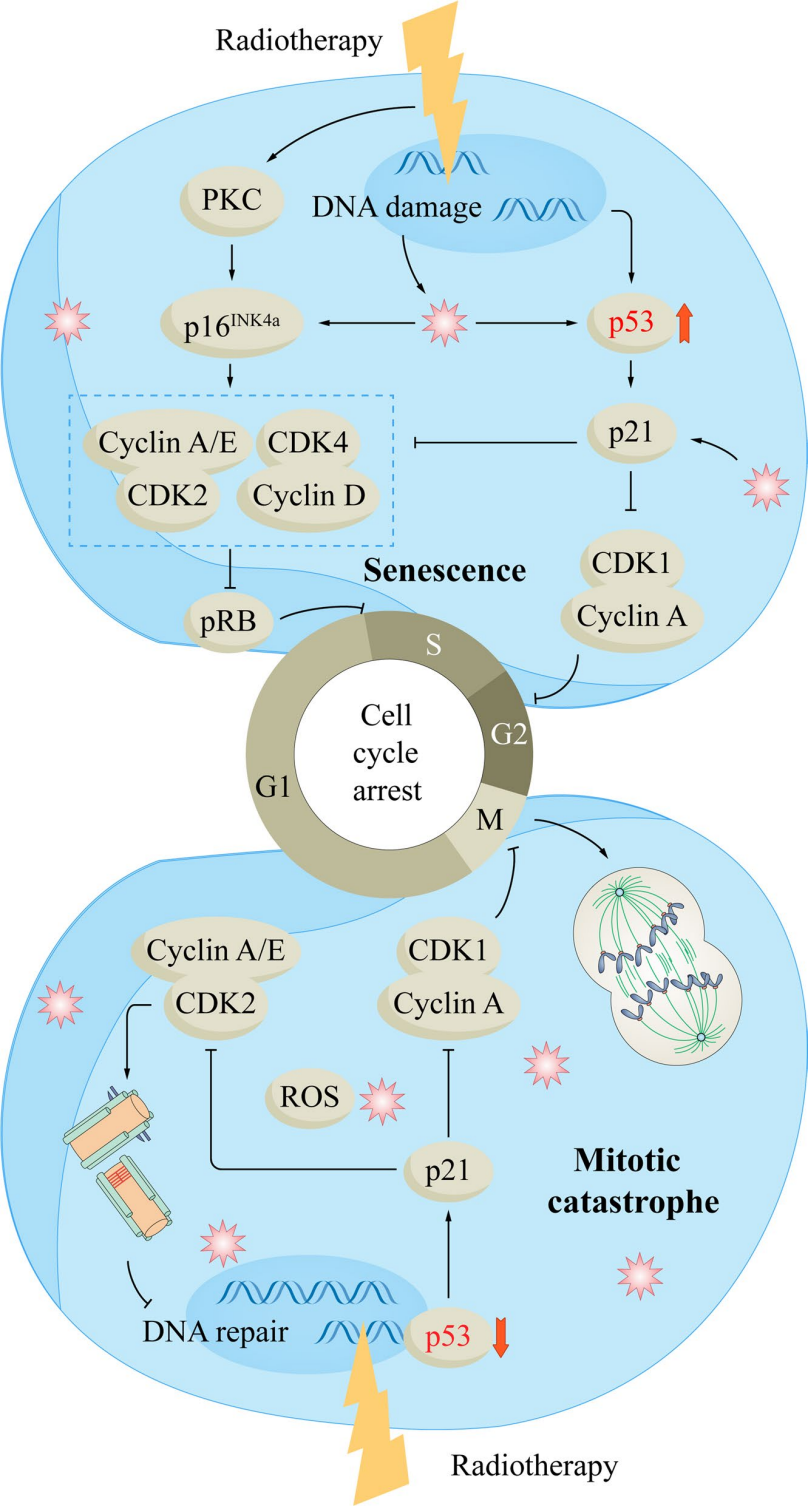
The mechanisms of RT-induced senescence and mitotic catastrophe in tumor cells. When p53 functions normally, ionizing radiation damages the DNA of tumor cells and activates the p53-p21 pathway and the p16INK4a-pRB pathway. The activation of p21 inhibits CDK1/2/4-cyclin complex, and the inhibition of CDK1-cyclin A complex arrests the cell cycle in the G2/M phase; p16INK4a inhibits CDK2-cyclin E/A complex and CDK4-cyclin D complex, promotes the dephosphorylation of pRB, and arrests the cell cycle in the G1/S phase, resulting in tumor cell senescence. When p53 is inactivated or mutated in tumor cells, ionizing radiation cannot activate the p53-p21 pathway, CDK1-cyclin A complex and CDK2-cyclin E/A complex are activated, and cells enter mitosis in advance; CDK2-cyclin E/A complex activation leads to centrosome hyperamplification. Tumor cells enter mitosis prematurely with unrepaired DNA damage, leading to mitotic catastrophe. CDK: cyclin-dependent kinase; pRB: retinoblastoma protein; RT: radiotherapy

When a mitotic catastrophe occurs, cells continue mitosis after a brief G2 arrest, and then the expression of multiple mitotic checkpoint proteins is increased and mitotic delay or arrest is promoted [114]. During this period, delayed apoptosis is activated in metaphase. Studies have shown that caspase-2 plays a key role in delayed apoptosis after mitotic catastrophe [115]. In general, however, cells do not die in metaphase because they can adapt to the mitotic checkpoint. At this time, the cells continue through one or more cell cycles and acquire an increasing number of chromosomal aberrations, becoming aneuploid or polyploid. Ultimately, these cells die due to delayed apoptosis or necrosis or enter cellular senescence [98]. It depends on the time of mitotic arrest, the capability of cells to exit from abnormal mitosis, and the activity of signaling pathways such as p53 and Hippo [98, 116, 117]. Studies have reported that delayed death after RT-induced mitotic catastrophe generally occurs 2–6 days after RT [118]. Cheng et al. found that 2 Gy RT could induce mitotic catastrophe in oral cancer cells in vitro and in vivo with B12536 [119]; Gordon et al. found that combined treatment of LB100 and protein phosphatase 2A could significantly reduce p53 expression, induce mitotic catastrophe, and increase the radiosensitivity of glioblastoma cells [120]. Figure 4 shows the mechanisms of RT-induced senescence and mitotic catastrophe in tumor cells.

## Radiotherapy and cuproptosis

Like iron, copper is also a trace element necessary for human survival. Copper is a cofactor for many essential enzymes in the body and is involved in various metabolic pathways, but high concentrations of copper can also lead to cell death [121].

In 2022, Tsvetkov et al. discovered a novel copper-dependent PCD and named it cuproptosis [122]. Their studies confirmed that ferredoxin 1 (FDX1) and protein acylation are key factors in the induction of cuproptosis [122]. FDX1 encodes a reductase that reduces Cu^2+^ to the more toxic Cu^+^. Cu^+^ is the target of the efficient copper ionophore elesclomol (ES) [122]. Studies have shown that ESs, such as disulfiram and NSC319726, can target tumor cells with high concentrations of copper [123]. Deletion of FDX1 leads to resistance to many copper ionophores.

In the tricarboxylic acid cycle (TCA), pyruvate dehydrogenase (PDH) complexes are acylated by key enzymes in the lipoic acid pathway (such as lipoyl synthase (LIAS)) and play important physiological functions [122]. Cu^+^ can directly bind to acylated proteins and promote their oligomerization and dysregulation, block TCA, trigger proteotoxic stress, and induce cuproptosis [122].

Studies have reported that RT could target copper metabolism in hepatocellular carcinoma, but the mechanism of RT-induced cell cuproptosis remains unclear [124, 125]. It has been determined that copper uptake in tumor cells is higher than that in normal cells, and in-depth research on the mechanism of radiation-induced cuproptosis will play an important role in developing methods to precisely kill tumor cells.

## Immunological consequences of RT-related cell fate decisions and tumor radiotherapy

RT can kill tumor cells by various CFDs, and then stimulate the immune response of the body to increase the therapeutic effect. Tumor suppresses the immune system during its growth and innate immunity and adaptive immunity both have anti-cancer effects [34]. The immune response activated by RT is related to the dose. Apoptosis is a cell death with low immunogenicity [11]. Apoptosis cells are engulfed by adjacent macrophages and presented to lymphocytes to promote immune response [12]. Necrosis and various PCDs with necrosis-like morphology are highly immunogenic cell death. Rupture of cell membrane and release of cell contents can promote the infiltration of inflammatory cells and immune cells, promote a stronger immune response, and alleviate the immunosuppression of the tumor microenvironment [34]. Compared with high-dose RT, the role of apoptosis and non-lethal processes is greater in low-dose RT, and senescence and mitotic catastrophe can also enter apoptosis [13, 98, 101]; In high-dose RT, necrosis-like cell death may play a greater role [13]. However, the response of immune cells to RT is also an important part to determine the effect of RT. Regulatory T cells (Treg) with immunosuppressive function are more sensitive to low-dose RT than CD4^+^ T and CD8^+^ T cells with immune response function; CD4^+^ T and CD8^+^ T cells are more sensitive to high-dose RT than Tregs, NK cells, and dendritic cells [126–128]. Studies have shown that low-dose RT was more effective in the local control of tumors, and had fewer side effects on normal cells and tissues [126, 129]. Therefore, apoptosis resistance of tumor cells is an important part of radioresistance, and also an important factor hindering the effect of RT. Because it reduces the effect of low-dose RT, the side effects of high-dose RT on immune cells and normal cells still need to be overcome.

## Conclusion

RT plays an important role in cancer treatment, and various cell fate decisions play an important role in tumor radiotherapy. Radiation-induced different cell fate decisions are related to various factors such as tumor cell line, tumor microenvironment, and radiation dose. However, studies that systematically discuss the conditions under which radiation induces different cell fate decisions are still lacking. For different tumors, especially various RT-resistant tumors, an in-depth understanding of the relevant cell fate decision mechanism will be helpful for the application of RT. On top of this, targeted therapy that modulates various cell fate decisions, such as targeting CFDs-related non-coding RNAs, will be an important therapy to increase the specificity and sensitivity of RT to tumor cells.

## Abbreviations

ACSL4: Acyl-CoA synthetase long-chain 4
Apaf-1: Apoptotic protease activating factor-1
ASC: Adaptor apoptosis-associated speck-like protein containing a CARD
Akt: Protein kinase B
CAD: Caspase-activated deoxyribonuclease
CDK: Cyclin-dependent kinase
CFD: Cell fate decision
cIAP: Cellular inhibitor of apoptosis protein
Cys2: Cystine
cyt-c: Cytochrome-c
DAMP: Damage-associated molecular pattern
DR: Death receptor
ER: Endoplasmic reticulum
FADD: Fas-associating protein with a novel death domain
GGC: γ-Glutamylcysteine
GSDM: Gasdermin
IKK: Inhibitor of NF-κB (IκB) kinase
LPCAT3: Lysophosphatidylcholine acyltransferase 3
MLKL: Mixed lineage kinase domain-like protein
MTORC1: Mechanistic target of rapamycin complex 1
N-PFD: N-terminal pore-forming domain
PCD: Programmed cell death
PI3K: Phosphatidylinositol 3-kinase
PLOOH: Phospholipid hydroperoxide
pRB: Retinoblastoma protein
PRR: Pattern recognition receptor
PUFA: Polyunsaturated fatty acid
PUFA-PL: Phospholipid containing polyunsaturated fatty acid chain
RIPK: Receptor-interacting serine/threonine-protein kinase
RT: Radiotherapy
SLC7A11: Solute carrier family 7 member 11
TRADD: TNF receptor-associated death domain
TRAF2: TNF receptor-associated factor 2
TRAIL2: TNF-related apoptosis-inducing ligand 2
